# Comparison of Fentanyl With Midazolam As Adjuvants to Levobupivacaine in Spinal Anesthesia for Cesarean Sections: A Randomized Controlled Trial

**DOI:** 10.7759/cureus.64732

**Published:** 2024-07-17

**Authors:** Shanmugam Yazhini, Rajagopalan Venkatraman, Karthik Kandan

**Affiliations:** 1 Anaesthesiology, SRM Medical College Hospital and Research Centre, Chennai, IND

**Keywords:** hemodynamics, postoperative nausea and vomiting, obstetrical, analgesia, midazolam, fentanyl, levobupivacaine, spinal, anaesthesia, caesarean section

## Abstract

Background and objectives

Spinal anesthesia stands as a cornerstone for patients undergoing lower segment cesarean section (LSCS), offering advantages like faster onset and high block density. Levobupivacaine, known for its high potency and long-acting nature, has a slower onset. The safety of intrathecal fentanyl or midazolam is evaluated as an adjuvant to levobupivacaine in parturients. This study aims to compare the duration of postoperative analgesia provided by fentanyl or midazolam added to 0.5% hyperbaric levobupivacaine in elective cesarean sections. Secondary objectives include evaluating the onset and duration of sensory and motor blockade and the incidence of nausea and vomiting. Identifying the more effective adjuvant will help optimize spinal anesthesia protocols, improve postoperative outcomes, and enhance patient comfort and recovery.

Methods

This study was conducted at SRM Medical College Hospital and Research Centre, Chennai, India, over six months (May 1, 2023, to October 1, 2023). A total of 90 patients undergoing elective LSCS received spinal anesthesia in a prospective randomized double-blinded controlled trial. Patients were allocated to three groups: Group A received levobupivacaine with fentanyl, Group B received levobupivacaine with midazolam, and Group C received levobupivacaine with normal saline. Block characteristics, postoperative analgesia, hemodynamic stability, and complications were assessed. Assessments were conducted at specified time points: intraoperatively, every five minutes for the first 30 minutes, every 10 minutes for the next hour, every two hours for six hours, and every four hours up to 24 hours postoperatively. Statistical analysis utilized one-way analysis of variance (ANOVA).

Results

Group B (levobupivacaine with midazolam) exhibited a shorter time to sensory block onset (88 seconds) compared to Groups A and C (both 145 seconds) (p < 0.001). Group A (levobupivacaine with fentanyl) showed a shorter time to maximum motor block (p = 0.045) than Groups B and C. The sensory block duration was significantly longer in Group A (127.5 minutes) compared to Group B (60 minutes) and Group C (69 minutes) (p < 0.001). Motor block duration was also prolonged in Group A (251 minutes) compared to Group B (147 minutes) and Group C (177 minutes) (p = 0.045). The first analgesic requirement was delayed in Group A (248 minutes), whereas Groups B (115 minutes) and C (90 minutes) (p < 0.001) required more frequent analgesia. Group A experienced a higher incidence of postoperative nausea and vomiting.

Conclusion

Midazolam accelerated sensory block onset, while fentanyl prolonged anesthesia duration without significantly affecting motor block. Fentanyl delayed the first analgesic requirement, whereas midazolam reduced postoperative nausea, vomiting, and shivering.

## Introduction

Administering intrathecal medications targeted at spinal cord receptors has been recognized for their ability to provide prolonged and superior-quality analgesia [1]. Anesthesiologists bear a moral imperative to ensure a safe and pain-free post-operative period, facilitating early patient ambulation and discharge through strategic medication and technique selection [1].

In obstetric anesthesia, there is a noticeable shift towards regional anesthesia (RA) over general anesthesia (GA) for cesarean sections, aligning with pregnant women's preference to remain conscious during delivery [2]. RA is favored for its perceived safety and reduced risk of drug-related complications [2].

Levobupivacaine, the S(−)-enantiomer of bupivacaine, is known for its favorable pharmacokinetic profile, providing effective surgical sensory block with outcomes comparable to conventional methods, ensuring safety for both mother and fetus [3,4]. Its preference in spinal anesthesia is due to lower cardiovascular side effects and reduced central nervous system toxicity compared to racemic bupivacaine [5-7], making it particularly advantageous for cesarean sections [8].

The addition of low doses of opioids to local anesthetics during spinal anesthesia for cesarean sections has shown promise in reducing local anesthetic-related side effects, shortening onset time, and improving intra- and post-operative analgesia quality by minimizing the required local anesthetic dose [9]. Fentanyl, a potent synthetic opioid, is preferred for its rapid onset and limited upward spread in subarachnoid anesthesia. Its combination with reduced bupivacaine doses enhances surgical anesthesia efficacy and block reliability [10]. Studies support its safety and efficacy, with dosages up to 25 mcg proving effective in cesarean sections [11].

Intrathecal midazolam, acting on spinal benzodiazepine receptors, synergistically enhances postoperative analgesia when combined with intrathecal bupivacaine [12]. Notably, the addition of 2 mg of midazolam to hyperbaric bupivacaine for elective cesarean sections has been shown to provide significant and effective postoperative analgesia [13]. Midazolam enhances gamma-aminobutyric acid (GABA)ergic currents via type A GABA receptors to induce analgesia [14]. Importantly, midazolam maintains stable intraoperative hemodynamics without compromising sensory and motor block levels, highlighting its safety and efficacy in obstetric anesthesia [15,16].

Elective cesarean sections provide a standardized and controlled environment for anesthesia research. The dosage of anesthetic agents is typically uniform, minimizing variability and allowing for a more precise assessment of the efficacy and safety of the adjuvants. In contrast, other surgical procedures often involve variable dosages due to differing complexities and durations, making it harder to standardize and compare results. Both fentanyl and midazolam have been used as adjuvants in various surgical settings, showing benefits in enhancing the quality of spinal anesthesia and providing postoperative analgesia. However, their comparative effectiveness and safety profile when used with levobupivacaine in cesarean sections, are not well-documented. The findings of this study will be directly applicable to emergency scenarios. High-risk patients, who are more prone to complications and may experience inadequate analgesia with standard protocols, will benefit from optimized and standardized anesthesia regimens. Improved understanding of the blockade effects and potential adverse events, will inform clinical practice, leading to better patient outcomes, reduced need for additional medications, and minimized risk of polypharmacy.

In reviewing the literature, numerous clinical studies have explored the intrathecal use of fentanyl and midazolam in various lower limb and abdominal surgeries with bupivacaine as the local anesthetic. Our study emphasis particularly highlights cesarean sections, focusing on fixed dosages to prevent discrepancies when incorporating levobupivacaine.

## Materials and methods

Following Institutional Ethics Committee approval, this study was registered with the Clinical Trial Registry - India (CTRI/2023/01/049315). It involved a prospective, double-blinded randomized study comprising 90 patients undergoing elective cesarean section at SRM Medical College Hospital and Research Centre, Chennai, India, over six months (May 1, 2023, to October 1, 2023). Written informed consent was obtained from all participants after counseling. The study's inclusion criteria comprised patients aged 18 to 35 years, classified as American Society of Anesthesiologists (ASA) II, and scheduled for elective cesarean section with a single intrauterine gestation. Exclusion criteria included significant cardiovascular or hepatorenal diseases, altered mental status, deranged coagulation profile, recent use of antiemetics within 24 hours, contraindications to central neuraxial blockade, and hypersensitivity to the study drug. All eligible patients underwent pre-anesthetic evaluation in the clinic, where they were thoroughly assessed and counseled prior to enrollment in the study.

Procedure

The study group included participants categorized as follows: Group A received 1.8 mL of 0.5% hyperbaric levobupivacaine and 20 mcg of fentanyl (0.4 mL); Group B received 1.8 mL of 0.5% hyperbaric levobupivacaine and 2 mg of preservative-free midazolam (0.4 mL). Preservative-free midazolam, utilized as an adjunct in spinal anesthesia, is available in our country in concentrations of 1 mg/mL and 5 mg/mL. For this study, we employed the 5 mg/mL concentration (Mezolam; Neon Laboratories Ltd., Mumbai, India), ensuring a preservative-free solution suitable for intrathecal use, where we administered 0.4 mL, equivalent to 2 mg of midazolam. Group C received 1.8 mL of 0.5% hyperbaric levobupivacaine with 0.4 mL of normal saline. Each patient underwent detailed counseling about the anesthesia procedure, including education on the Visual Analog Scale (VAS), and explicit informed consent was obtained. Relevant details such as age, weight, ASA grade, and duration of the surgery were documented.

Upon entry into the operating theatre, patients were connected to a multipara monitor to record vital signs: heart rate (HR), respiratory rate (RR), oxygen saturation (SpO2), non-invasive blood pressure (NIBP), and electrocardiogram (ECG). An 18G IV cannula was inserted, and preload intravenous fluid (Ringer lactate solution) was administered at 10 mL/kg. Baseline parameters, including HR, RR, systolic blood pressure (SBP), diastolic blood pressure (DBP), mean arterial pressure (MAP), and SpO2, were measured. Continuous ECG monitoring with a three-lead display was initiated.

Premedication included 0.25 mg of alprazolam, 150 mg of ranitidine, and 10 mg of metoclopramide, administered on the day before and the morning of the surgery with sips of water. A Foley catheter was inserted for urine output monitoring. Spinal anesthesia was administered using a 25-/26-gauge Quincke’s spinal needle in the L3-4/L4-5 intervertebral space, followed by slow injection of 0.1 mL/sec into the subarachnoid space, ensuring free cerebrospinal fluid flow. Patients were then positioned supine with shoulder support.

The sensory block was assessed using the pinprick method, by assessing bilaterally in the midclavicular line with a short beveled 25-gauge sterile needle and a cotton swab. Care was taken to compare sides and segments systematically, ensuring accuracy without penetrating the dermis, while the quality of the motor block was evaluated using the modified Bromage scale, prior to surgery. Intraoperative parameters recorded included sensory block onset, duration of sensory block, and motor block duration. Incidences of nausea and vomiting were noted. Hemodynamic parameters were noted at specified intervals, and any adverse reactions were documented. The neonatal APGAR score will be recorded at the first- and fifth-minute of delivery.

The modified Bromage scale assesses motor block [4]: Bromage 0 means the patient can move the hip, knee, and ankle and lift their leg against gravity. Bromage 1 signifies an inability to lift the leg against gravity, but the ability to flex the knee and ankle. Bromage 2 indicates an inability to flex the hip and knee, but the ability to flex the ankle. Bromage 3 means inability to flex the hip, knee, and ankle, but the ability to move the toes, while Bromage 4 indicates complete paralysis.

The Hollmen’s scale [4] classifies sensory responses during spinal anesthesia: Grade 0 indicates normal sensation to pinprick, Grade 1 describes a weaker sensation compared to the opposite side, Grade 2 indicates feeling touch with a blunt object, and Grade 3 denotes no perception of the pinprick.

The Nausea Vomiting Grade scale [15] categorizes symptoms: Grade 0 denotes no symptoms, Grade 1 indicates nausea, Grade 2 signifies retching, and Grade 3 represents vomiting.

The study evaluates several parameters. Firstly, the duration of postoperative analgesia was determined based on the time of the first analgesic requirement following surgery, and subsequent assessment of pain using the VAS. Secondly, the onset of the sensory blockade was recorded as the time taken to achieve the level of the Grade 3 Hollmans scale, with no sensory response using a pinprick test at the level of T6. Thirdly, the duration of both sensory and motor blocks was recorded: sensory block duration includes the time from the peak of sensory block up to two sensory regressions, or when the patient feels pain in the field of surgery, while motor block duration encompasses the time from maximum motor block intensity until achieving a Bromage Grade 1 score. Lastly, the study will document the incidence of nausea and vomiting postoperatively, focusing on these occurrences as observed parameters.

Postoperatively, pain intensity and quality were assessed using the VAS scale. The time to first rescue analgesia and any adverse reactions were recorded. Analgesics, such as Inj. paracetamol 1 g IV, was administered when the VAS score was ⥸ 3. Inj. paracetamol was repeated every sixth hour for 24 hours. If the patient still complained of pain (VAS score ⥸ 3), Inj. ketorolac 30 mg was given. Fluid administration, blood loss calculations, and administration of medications like ondansetron and tramadol were done as necessary. Total consumption of analgesics was recorded. The VAS score was recorded every fourth hour for 24 hours.

Following surgery, patients were shifted to the postoperative ward. Hemodynamic monitoring, such as HR, BP, and saturation, was continuously monitored throughout the surgery and for 24 hours postoperatively. These parameters were recorded every five minutes for the first 30 minutes, then every 10 minutes for the next hour, followed by every two hours for the subsequent six hours, and every four hours until 24 hours postoperatively.

Data analysis

Descriptive statistics were utilized to analyze the data, encompassing measures such as mean, median, standard deviation, and frequency distributions. Each study group required a sample size of 30 patients, achieving a statistical power of 95% with a significance level (alpha error) of 5%. Data were meticulously collected, tabulated, and statistically analyzed using IBM SPSS Statistics for Windows, Version 20 (Released 2011; IBM Corp., Armonk, NY, USA). Quantitative data were presented as mean ± SD, while qualitative data were expressed as percentages and counts. Statistical significance was defined as a p-value < 0.05. Associations between categorical variables were examined using the Chi-square test or Fisher's exact test, and differences between continuous variables were assessed using Student’s t-test. This methodological approach ensures rigorous analysis and robust interpretation of findings in the study.

## Results

**Figure 1:**
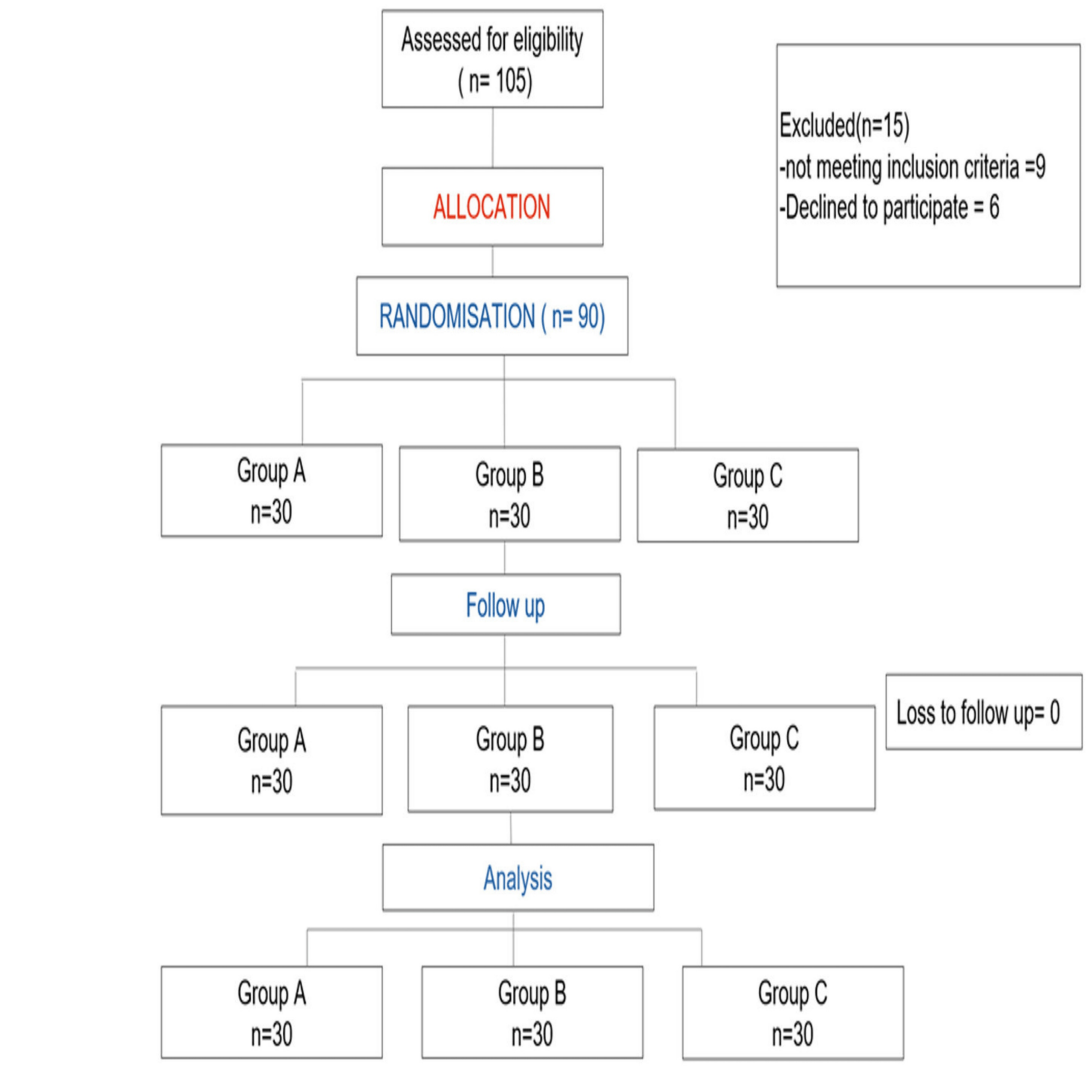
Consort diagram

A total of 90 patients were randomized, with 30 patients in each group, as shown in the consolidated visual representation depicting the passage of participants through this study (Figure 1).

As shown in Table 1, the distribution of mean age, weight, and duration of surgery across all three groups indicates no significant differences when comparing the average age among the groups (Group A: 26.97 ± 4.69 years, Group B: 25.93 ± 3.19 years, Group C: 26.43 ± 2.86 years; p = 0.55). This indicates comparable age profiles across the groups. Similarly, there were no statistically significant differences in BMI (Group A: 24.53 ± 2.34 kg/m², Group B: 23.76 ± 3.33 kg/m², Group C: 24.43 ± 3.77 kg/m²; p = 0.60) or duration of surgery (Group A: 128.66 ± 24.99 minutes, Group B: 128 ± 21.96 minutes, Group C: 122 ± 21.55 minutes; p = 0.46) among the groups, confirming comparability in these aspects as well. 

**Table 1:** Mean age, weight, and duration of surgery of study participants (n = 90) Data are expressed as mean ± SD or as the number of patients; the p-value is significant if p < 0.05, so here the p-value is not significant NS: Not significant

Parameter	Group A	Group B	Group C	p-value (Groups A vs. B vs. C)
Age (years)	26.97 ± 4.69	25.93 ± 3.19	26.43 ± 2.86	0.55 (NS)
BMI (kg/m^2^)	24.53 ± 2.34	23.76 ± 3.33	24.43 ± 3.77	0.60 (NS)
Duration of surgery (minutes)	128.66 ± 24.99	128 ± 21.96	122 ± 21.55	0.46 (NS)

**Figure 2:**
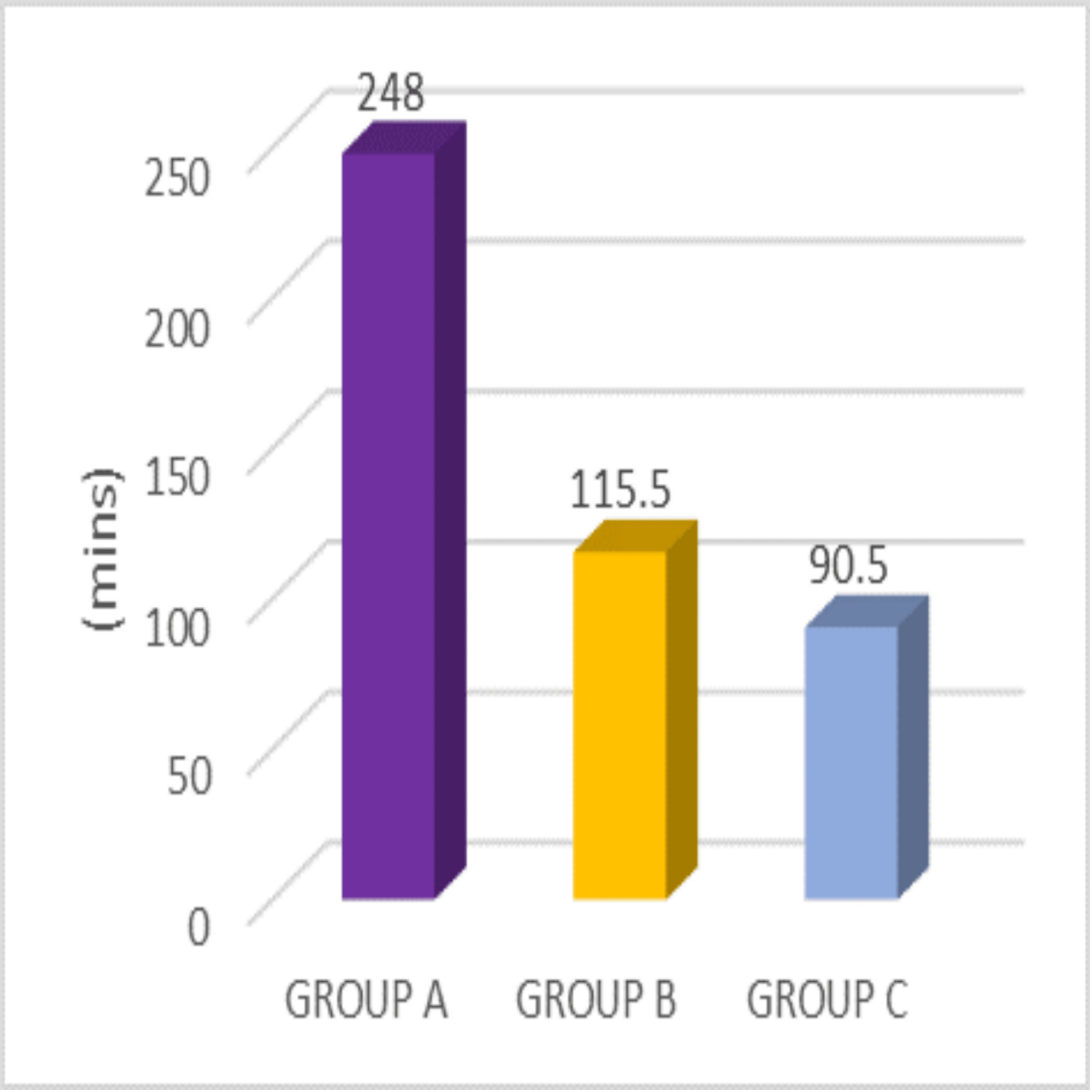
Duration of first analgesia requirement (minutes) in Groups A, B, and C with different adjuncts to levobupivacaine

Here we describe the first requirement of analgesia of the participants in the three groups. The findings reveal a notable statistical difference among the three groups, supported by a p-value of <0.001. It's noteworthy that Group C (90.5 ± 53.36) required analgesia earlier than Group B (115.5 ± 39.2), while Group B required an analgesic dose earlier than Group A (248 ± 78) (p-value < 0.001), indicating a distinct trend in analgesic need across the groups, as shown in Figure 2.

**Figure 3:**
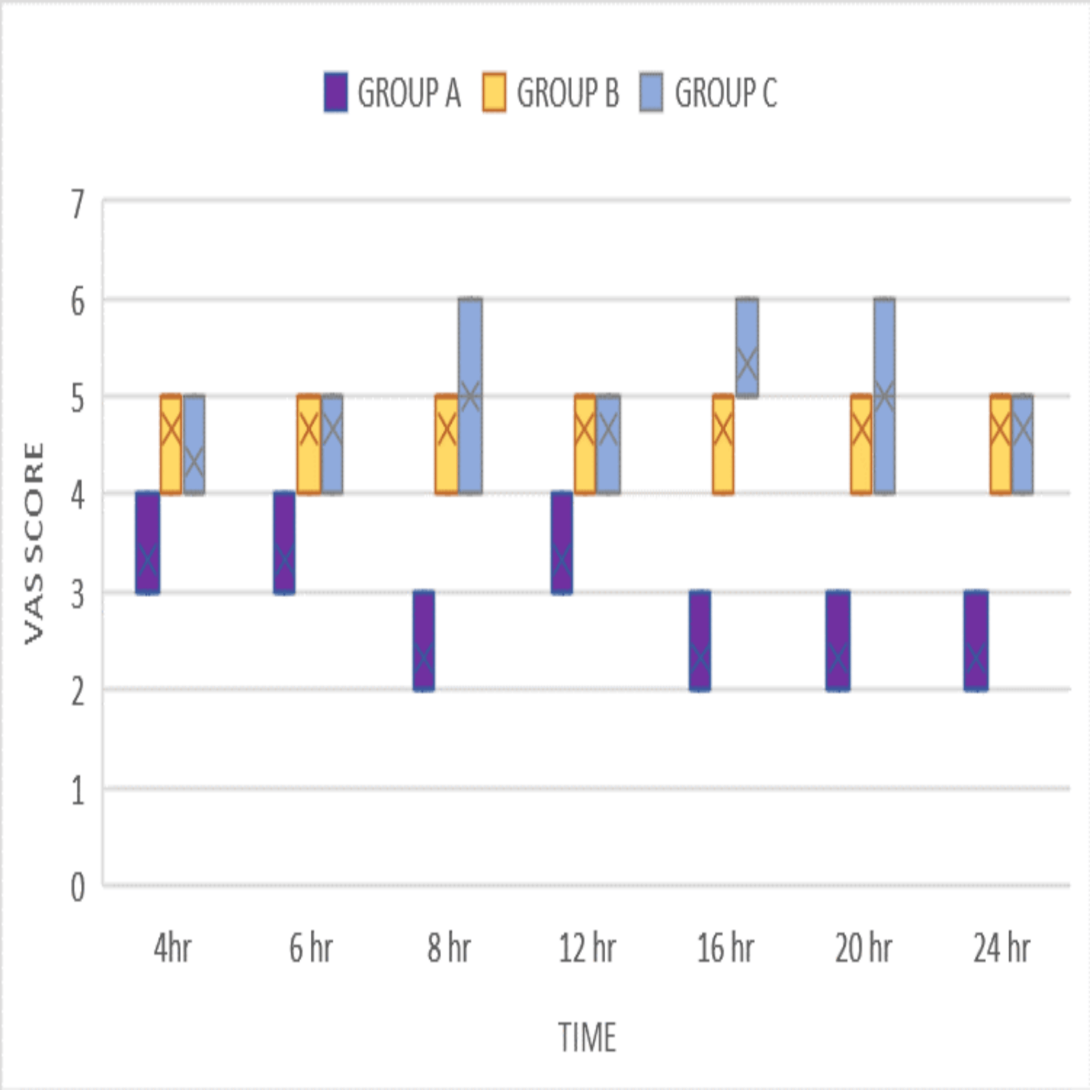
VAS scores at different time points in Groups A, B, and C with different adjuncts to levobupivacaine VAS: Visual analog score

Table 2 presents the interquartile median range of VAS scores at various time points. Group A shows significantly better VAS scores compared to both Group B and Group C (p < 0.001), indicating statistical significance. However, there is no statistically significant difference between Group B and Group C, as depicted in Figure 3.

**Table 2:** VAS score at different time points (n = 90) Data are expressed as median, or as number of patients; *p-value is significant (p < 0.05) VAS: Visual analog score; NS: Not significant

Time (hr)	Group A	Group B	Group C	p-value (Group A vs. Group B)	p-value (Group B vs. Group C)	p-value (Group A vs. Group C)
4	3 (4-3)	5 (5-4)	4 (5-4)	<0.001*	0.74 (NS)	<0.001*
6	3 (4-3)	5 (5-4)	5 (5-4)	<0.001*	0.62 (NS)	<0.001*
8	2 (3-2)	5 (5-4)	5 (6-4)	<0.001*	0.41 (NS)	<0.001*
12	3 (4-3)	5 (5-4)	5 (5-4)	<0.001*	1.0 (NS)	<0.001*
16	2 (3-2)	5 (5-4)	5 (6-5)	<0.001*	0.15 (NS)	<0.001*
20	2 (3-2)	5 (5-4)	5(6-4)	<0.001*	0.58 (NS)	<0.001*
24	2 (3-2)	5 (5-4)	5 (5-4)	<0.001*	0.90 (NS)	<0.001*

At four hours, the interquartile median range in Group A is 3 (4-3). In Group B, the interquartile range is 5 (5-4). In Group C, the interquartile range is 4 (5-4). The p-value is <0.001 overall. In intergroup comparisons, the p-value is <0.001 for Group A vs. Group B and Group A vs. Group C, while it is 0.74 for Group B vs. Group C.

At six hours, the interquartile median range in Group A is 3 (4-3). In Group B, the interquartile range is 5 (5-4). In Group C, the interquartile range is 5 (5-4). The p-value is <0.001 overall. In intergroup comparisons, the p-value is <0.001 for Group A vs. Group B and Group A vs. Group C, while it is 0.62 for Group B vs. Group C.

At eight hours, the interquartile median range in Group A is 2 (3-2). In Group B, the interquartile range is 5 (5-4). In Group C , the interquartile range is 5 (6-4). The p-value is <0.001 overall. In intergroup comparisons, the p-value is <0.001 for Group A vs. Group B and Group A vs. Group C, while it is 0.41 for Group B vs. Group C.

At 12 hours, the interquartile median range in Group A is 3 (4-3). In Group B, the interquartile range is 5 (5-4). In Group C, the interquartile range is 5 (5-4). The p-value is <0.001 overall. In intergroup comparisons, the p-value is <0.001 for Group A vs. Group B and Group A vs. Group C, while it is 1.0 for Group B vs. Group C.

At 16 hours, the interquartile median range in Group A is 2 (3-2). In Group B, the interquartile range is 5 (5-4). In Group C, the interquartile range is 5 (6-5). The p-value is <0.001 overall. In intergroup comparisons, the p-value is <0.001 for Group A vs. Group B and Group A vs. Group C, while it is 0.15 for Group B vs. Group C.

At 20 hours, the interquartile median range in Group A is 2 (3-2). In Group B, the interquartile range is 5 (5-4). In Group C, the interquartile range is 5 (6-4). The p-value is <0.001 overall. In intergroup comparisons, the p-value is <0.001 for Group A vs. Group B and Group A vs. Group C, while it is 0.58 for Group B vs. Group C.

At 24 hours, the interquartile median range in Group A is 2 (3-2). In Group B, the interquartile range is 5 (5-4). In Group C, the interquartile range is 5 (5-4). The p-value is <0.001 overall. In intergroup comparisons, the p-value is <0.001 for Group A vs. Group B and Group A vs. Group C, while it is 0.90 for Group B vs. Group C.

**Figure 4:**
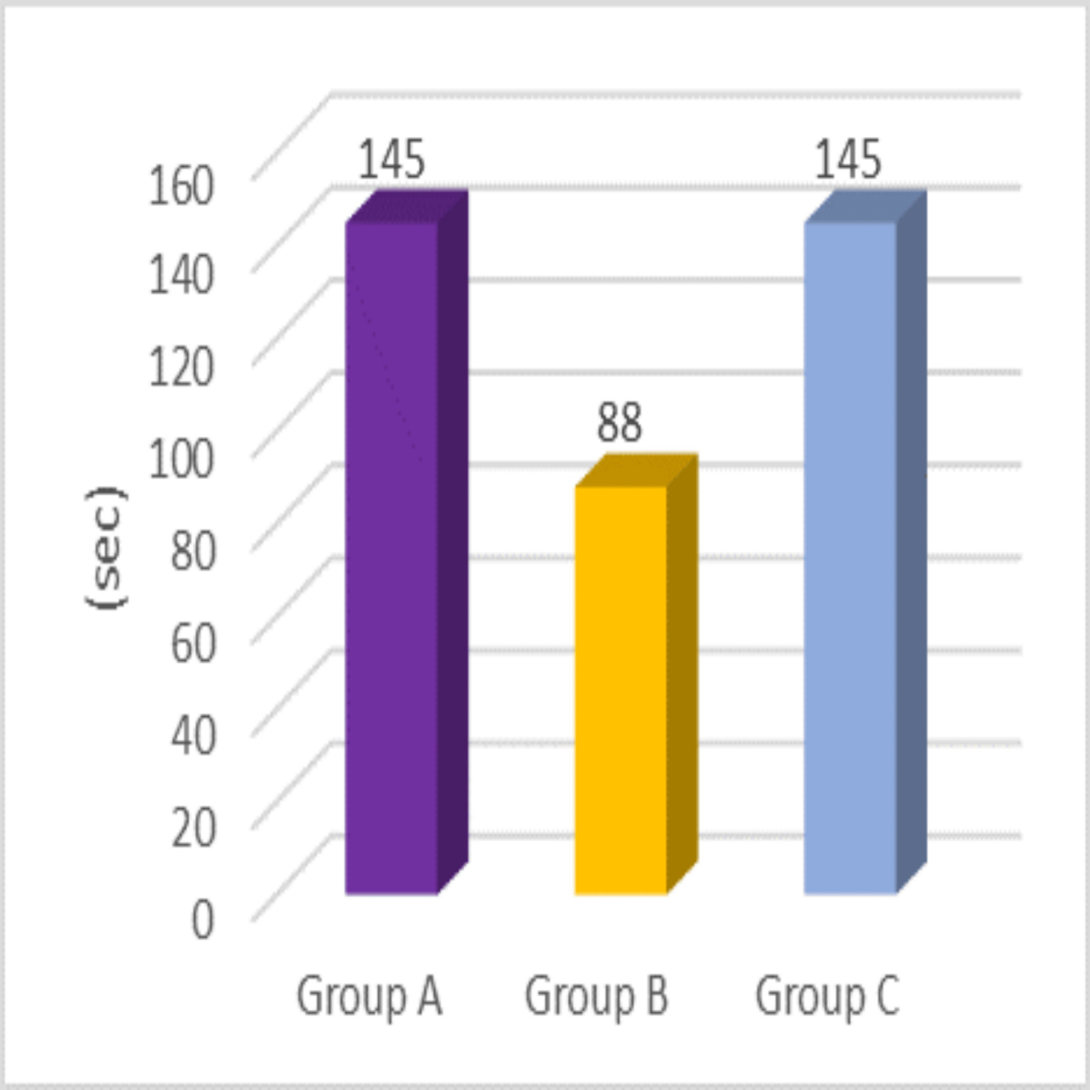
Onset of sensory blockade in Groups A, B, and C with different adjuncts to levobupivacaine

In Figure 4, the time of onset of sensory block was 145 ± 49.88 seconds for Group A, 88 ± 27.22 seconds for Group B, and 145 ± 38.6 seconds for Group C. The difference in these times is statistically significant (p < 0.001).

**Figure 5:**
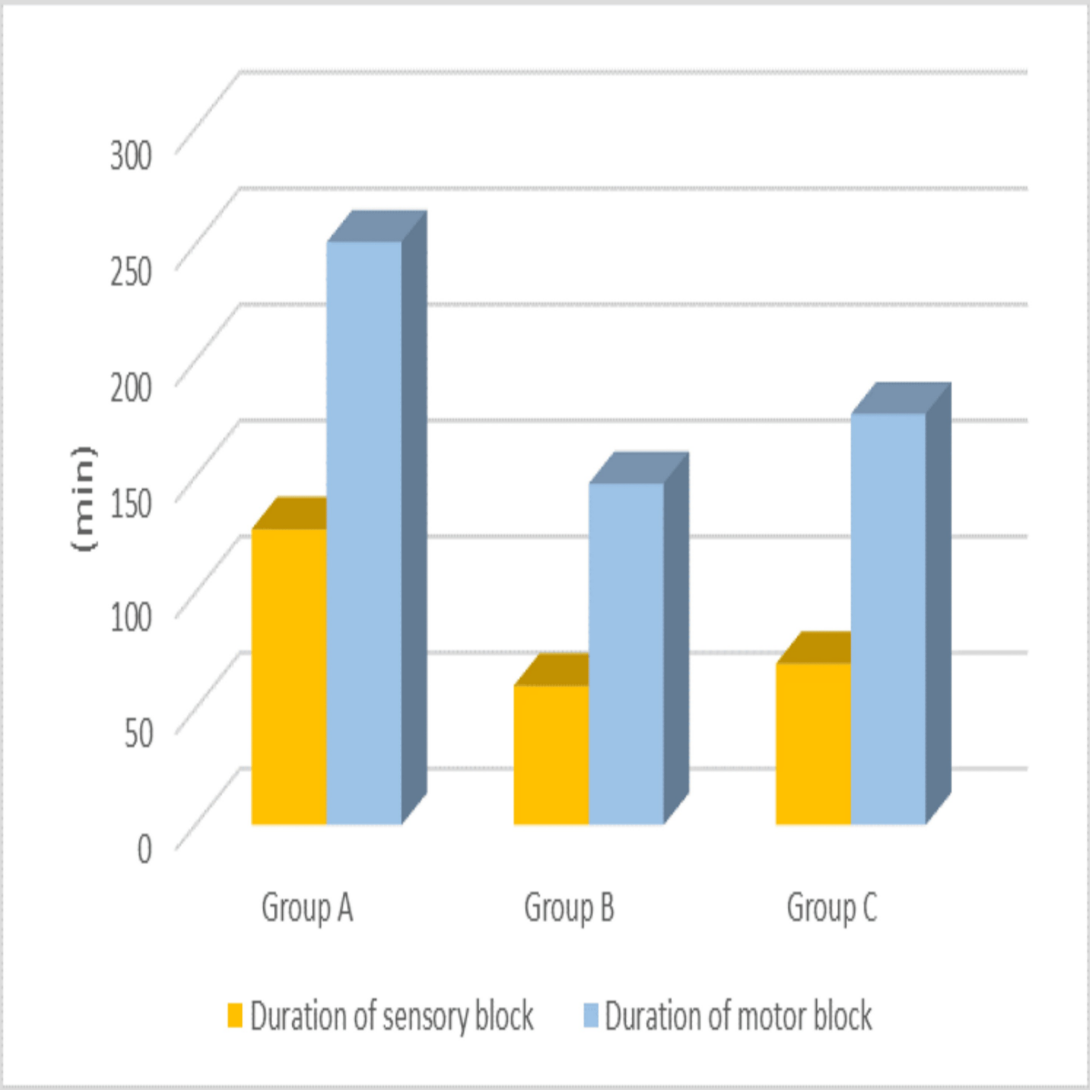
Duration of sensory and motor blockade in Groups A, B, and C with different adjuncts to levobupivacaine

The duration for achieving maximum motor block was as follows: Group A required 251 ± 11.39 minutes, Group B required 147 ± 9.31 minutes, and Group C required 177 ± 31.69 minutes (p < 0.001). The time for two-segment regression was 127.50 ± 26.35 minutes for Group A, 60 ± 22.63 minutes for Group B, and 69.50 ± 18.26 minutes for Group C (p < 0.001), demonstrating statistical significance. This is also visually represented in Figure 5.

Table 3 describes the incidence of nausea and vomiting as follows: Group A had four patients with grade 1 symptoms, Group C had two patients with grade 1 symptoms, and Group B had no reported cases. However, there was no statistically significant difference observed, even when comparing between the groups.

**Table 3:** Incidence of nausea and vomiting (n = 90) Data are expressed as mean ± SD or as a number of patients; the p-value is significant if p < 0.05, here the p-value is not significant PONV: Postoperative nausea vomiting; NS: Not significant

PONV	Group A	Group B	Group C	p-value (Group A vs. Group B)	p-value (Group B vs. Group C)	p-value (Group A vs. Group C)
Grade 0	26	30	28	0.11 (NS)	0.49 (NS)	0.67 (NS)
Grade 1	4	0	2			
Grade 2	0	0	0			
Grade 3	0	0	0			

Table 4 details the mean requirement of rescue analgesics. In Group A, the mean requirement for Inj. paracetamol was 2.87 ± 0.63, significantly lower compared to Group B (7.30 ± 0.65) and Group C (7.57 ± 0.77) (p < 0.001). Intergroup comparisons showed p < 0.001 for Group A vs. Group B and Group A vs. Group C, while the comparison between Group B and Group C yielded a p-value of 0.41. Similarly, for Inj. ketorolac, Group A had a mean requirement of 1.53 ± 0.52, significantly less than Group B (2.47 ± 0.57) and Group C (2.97 ± 0.56) (p < 0.001). Intergroup comparisons revealed p < 0.001 for Group A vs. Group B and Group A vs. Group C, and p = 0.002 for Group B vs. Group C.

**Table 4:** Rescue analgesics (n = 90) Data are expressed as mean ± SD; *p-value is significant if p < 0.05 NS: Not significant

Parameter	Group A	Group B	Group C	p-value (Groups A vs. B)	p-value (Groups B vs. C)	p-value (Groups A vs. C)
Paracetamol	2.87 ± 0.63	7.30 ± 0.65	7.57 ± 0.77	<0.001*	0.41 (NS)	<0.001*
Ketorolac	1.53 ± 0.52	2.47 ± 0.57	2.97 ± 0.56	<0.001*	0.002 (NS)	<0.001*

## Discussion

Regarding postoperative analgesia, previous studies have shown that fentanyl demonstrates superior analgesic efficacy over midazolam as an intrathecal adjuvant, albeit in studies that lacked a placebo group for comparison and had no uniform distribution of adjuvants across treatment groups [1,2,8,17]. However, in our study, we compared three distinct groups with a control group, revealing that the addition of normal saline to levobupivacaine necessitated earlier administration of the first analgesic dose compared to both midazolam and fentanyl. Notably, the midazolam group required earlier intervention than the fentanyl group.

Regarding the safety of midazolam, numerous studies conducted across India using preservative-free midazolam have consistently reported no significant or life-threatening side effects in various surgical procedures. Animal studies investigating midazolam's safety, particularly concerning potential neuropathological symptoms, have been conducted. To address this, a study involving humans was carried out to assess neuropathological symptoms with and without intrathecal midazolam. The findings revealed no adverse events compared to conventional therapy, affirming its safety. Patient safety, informed consent, and adherence to professional standards were rigorously maintained.

In contrast to some existing studies [2,18], which found no significant distinction between the midazolam group and control in terms of analgesic requests, our results contradicted this, showing that the addition of midazolam to bupivacaine led to a delayed requirement for rescue analgesia compared to the normal saline group [12,19]. Notably, there were no studies available examining the difference between combinations with control groups in addition to levobupivacaine.

Patients administered hyperbaric levobupivacaine 3 mL exhibited a prompt onset of sensory and motor block, achieving T4 sensory levels rapidly, which was deemed adequate for the planned surgical interventions [20]. However, this effect gradually waned approximately one hour post-spinal injection. Interestingly, our study demonstrated that the inclusion of adjuncts helped extend the duration of the block with a reduced dosage of levobupivacaine (1.8 mL), in contrast to previous findings [20].

Furthermore, while prior research suggested lower VAS scores in the midazolam group compared to the fentanyl group [21], our study revealed lower VAS scores in the fentanyl group compared to both the midazolam and control groups, with no statistically significant difference between midazolam and control. Additionally, the duration of postoperative analgesia was longest in the fentanyl group, followed by the midazolam group, and then the control group. Importantly, rescue analgesic usage was significantly lower in the fentanyl group compared to the midazolam and control groups. However, some studies contradicting our findings have mentioned that there is no significant difference in VAS scores between midazolam and fentanyl [22,23].

Regarding the onset of sensory blockade, previous studies indicated a faster onset with midazolam compared to fentanyl [1,18,24]. Our findings aligned with this, showing that adding midazolam to levobupivacaine resulted in a faster onset of sensory blockade compared to both fentanyl and normal saline.

Moreover, the duration of sensory and motor blockade was longer with fentanyl compared to midazolam and normal saline, with no significant difference observed between midazolam and the control group. This contrasts with some studies suggesting better analgesic quality with midazolam than fentanyl and a longer duration before the first analgesic request with midazolam [12,19,25], whereas some studies show no significant difference between onset and duration [22,26].

Regarding adverse effects, the incidence of nausea and vomiting did not significantly differ among groups, although a few participants reported these symptoms in the fentanyl group, while none did in the midazolam group. Contrary to our findings, a study demonstrated a lower incidence of nausea and vomiting in the group receiving a combination of intrathecal fentanyl compared to the group receiving intrathecal midazolam, albeit utilizing a lower fentanyl dosage of 12.5 mcg [15]. Nonetheless, several studies corroborate our results [16,27], indicating a low incidence of nausea and vomiting in the midazolam group. Notably, adjunct intrathecal midazolam has been suggested to offer potentially prolonged analgesia compared to opioids alone, while concurrently attenuating their adverse effects, including nausea and vomiting [28,29]. The speculated mechanism underlying the anti-emetic effect of benzodiazepines involves their action at the chemoreceptor trigger zone, thereby reducing the synthesis, release, and postsynaptic effect of dopamine [30].

Finally, no significant differences were observed in hemodynamics or neonatal outcomes between the midazolam and fentanyl groups, affirming the safety profile of both agents in this context.

Limitation 

Assessment of recovery and ambulation postoperatively was not within the scope of our investigation. Moreover, our study was conducted at a single center with a relatively smaller sample size, homogeneity of the patient population, and was restricted to ASA II patients aged 18-35 undergoing elective cesarean sections, further limiting generalizability. Additionally, the exclusion criteria, such as excluding patients with significant comorbidities, may limit applicability to diverse patient groups. Moving forward, exploring the efficacy of alternative adjuvants could offer valuable insights into refining spinal anesthesia techniques for improved patient outcomes.

## Conclusions

In this prospective randomized study, we evaluated the efficacy of intrathecal fentanyl (25 mcg) and midazolam (2 mg) as adjuncts to 0.5% hyperbaric levobupivacaine in parturients undergoing elective cesarean section under spinal anesthesia. Our results indicate that intrathecal fentanyl significantly prolonged postoperative analgesia compared to midazolam, while midazolam exhibited a faster onset of sensory block compared to fentanyl. Additionally, fentanyl provided a longer duration of both sensory and motor blockade. Both additives showed minimal effects on vital parameters such as HR, RR, SBP, DBP, MAP, SpO2, and ECG. Importantly, neither fentanyl nor midazolam exhibited significant adverse effects on monitored vital parameters. These findings suggest that both fentanyl and midazolam are safe and effective choices as adjuvants to 0.5% hyperbaric levobupivacaine, providing enhanced postoperative pain relief with minimal side effects. Specifically, fentanyl exhibited superior block duration, reduced analgesic requirements, and lower VAS scores compared to both midazolam and the control group.
